# A Synergistic Complexation‐Encapsulation Paradigm Enables Unprecedented Radioactive Remediation

**DOI:** 10.1002/advs.202504940

**Published:** 2025-10-03

**Authors:** Qilong Tang, Huaixin Hao, Xue Dong, Chao Xu, Zhipeng Wang

**Affiliations:** ^1^ Institute of Nuclear and New Energy Technology Tsinghua University Beijing 100084 P. R. China; ^2^ The 404 Company Limited China National Nuclear Corporation Lanzhou 732850 P. R. China

**Keywords:** americium, complexation‐encapsulation, eutectic mixtures, micelle, radioactive decontamination

## Abstract

The escalating global warming and surging energy demands have accelerated the pursuit of low‐carbon, stable, and high‐density nuclear energy as a sustainable alternative to conventional fossil fuels. However, nuclear energy deployment inevitably generates radioactive contamination, posing persistent risks to ecosystems and human health. The effective removal of radioactive pollutants remains a critical yet unresolved challenge. In this study, a novel and straightforward eutectic mixture‐based detergent is presented to address this issue. Leveraging an innovative synergistic mechanism integrating complexation and encapsulation, rapid and highly efficient decontamination of americium (Am), a potent α‐emitter, is achieved. Additionally, this detergent exhibits exceptional versatility, effectively removing radionuclides across a broad spectrum of oxidation states (+I to +VII). These results highlight its significant potential for practical application in radioactive remediation.

## Graphical abstract

A novel HDEHP‐APG eutectic mixture‐based detergent is developed that achieves highly efficient decontamination of Am through a synergistic complexation‐encapsulation mechanism. Remarkably, such a detergent effectively removes radionuclides across a wide range of oxidation states (+I to +VII), demonstrating exceptional versatility. These results highlight its significant potential for practical radioactive remediation applications.

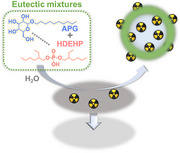

## 1 Introduction

Since the advent of the nuclear industry in the 1940s, the expansive demand for decommissioning nuclear facilities contaminated with radionuclides has emerged as a critical global concern.^[^ ^1^, ^2^ ^]^ This urgency is further compounded by catastrophic nuclear incidents, including the Chernobyl disaster and Fukushima Daiichi accident, which have highlighted the persistent challenges of surface radioactive contamination.^[^ ^3^, ^4^ ^]^ Accordingly, effective decontamination of radioactive pollution is critical for nuclear security and closely associated with the sustainable development of nuclear energy. In this context, foam‐based decontamination has garnered great attention due to its inherent advantages of less final waste volume, enhanced visual monitoring capabilities, cost‐effectiveness, superior surface adherence, and remote operation feasibility,^[^ ^5^, ^6^, ^7^, ^8^, ^9^, ^10^ ^]^ which dissolves the superficial pollutants and enables efficient transport of radioactive liquids through its exceptional mobility.^[^ ^7^, ^8^, ^11^ ^]^ Nevertheless, conventional foam detergents, predominantly formulated with water and traditional surfactants such as alkyl polyglucoside (APG),^[^ ^6^, ^9^, ^10^, ^12^, ^13^ ^]^ anionic surfactant (AOS)^[^ ^10^, ^13^, ^14^, ^15^, ^16^, ^17^ ^]^ and aliphatic acid (AA)^[^ ^18^, ^19^, ^20^ ^]^ exhibit poorly physical adsorption at the gas‐liquid interface, resulting in unsatisfied decontamination efficiency (*DE*).^[^ ^5^, ^6^, ^7^, ^8^, ^9^, ^10^, ^13^, ^17^, ^21^ ^]^ Moreover, present studies have predominantly focused on the remediation of weakly adhering β/γ‐emitting radionuclides, including cesium‐137 (^137^Cs),^[^ ^8^, ^22^, ^23^, ^24^, ^25^, ^26^ ^]^ strontium‐90 (^90^Sr),^[^ ^8^ ^]^ cobalt‐60 (^60^Co) ions^[^ ^17^, ^21^, ^27^ ^]^ and weakly α‐emitting isotopes of uranium‐235/238 (^235,238^U),^[^ ^7^, ^9^, ^10^, ^28^, ^29^, ^30^, ^31^, ^32^, ^33^, ^34^, ^35^ ^]^ neglecting the most critical α‐emitter americium‐241 (^241^Am).^[^ ^36^, ^37^, ^38^, ^39^ ^]^ Although great efforts have been devoted to enhancing foam stability and anti‐freezing properties through complex doping strategies, these approaches often compromise system homogeneity, increase operational costs, and hinder large‐scale implementation.^[^ ^5^, ^6^, ^10^, ^13^, ^17^ ^]^ Consequently, the development of a simple, stable, and universally applicable detergent system for efficient radioactive remediation remains an urgent scientific and technological imperative.

The structural stability and detergency efficacy of a specific foam are fundamentally governed by its interfacial properties, which can be strategically optimized through meticulous molecular design and precise modification of surfactant architecture. Such enhancements are predicated on leveraging intricate intermolecular chemical interactions rather than mere physical reagents integration. Fortunately, we identified that one of the aforementioned surfactants, APG, is a promising surfactant candidate, characterized by a hydrophilic terminus replete with hydroxyl functionalities. This structural motif has inspired us to modulate its properties via self‐assembled hydrogen bonding networks, employing complementary complexing agents as hydrogen bond acceptors (HBA) or donors (HBD). The resultant molecular ensembles can be classified as eutectic mixtures,^[^ ^40^, ^41^ ^]^ a class of materials that have found extensive utility in diverse domains, including metallurgy, synthetic chemistry, gas capture, energy storage systems, and pharmaceutical sciences,^[^ ^42^ ^]^ yet remain conspicuously underexplored in radiological decontamination.^[^ ^28^ ^]^ We anticipate that these materials will exhibit superior performance compared to traditional decontaminants out of the following considerations. First, powerful chemical interactions are expected to substantially enhance the surface characteristics of the detergent, thereby modulating its fundamental wetting behavior. Second, the formation of intricate hydrogen bonding networks within the molecular architecture portends a marked improvement in the mechanical integrity of the detergent system. Third, the incorporation of specialized complexing agents facilitates decontamination efficacy through optimized coordination chemistry. Fourthly, in contrast to conventional APG‐based systems, this synthetic paradigm potentially engenders a fundamentally distinct mechanism of pollutant removal, paving the way for a more efficient and sophisticated decontamination methodology.

**Figure 1:**
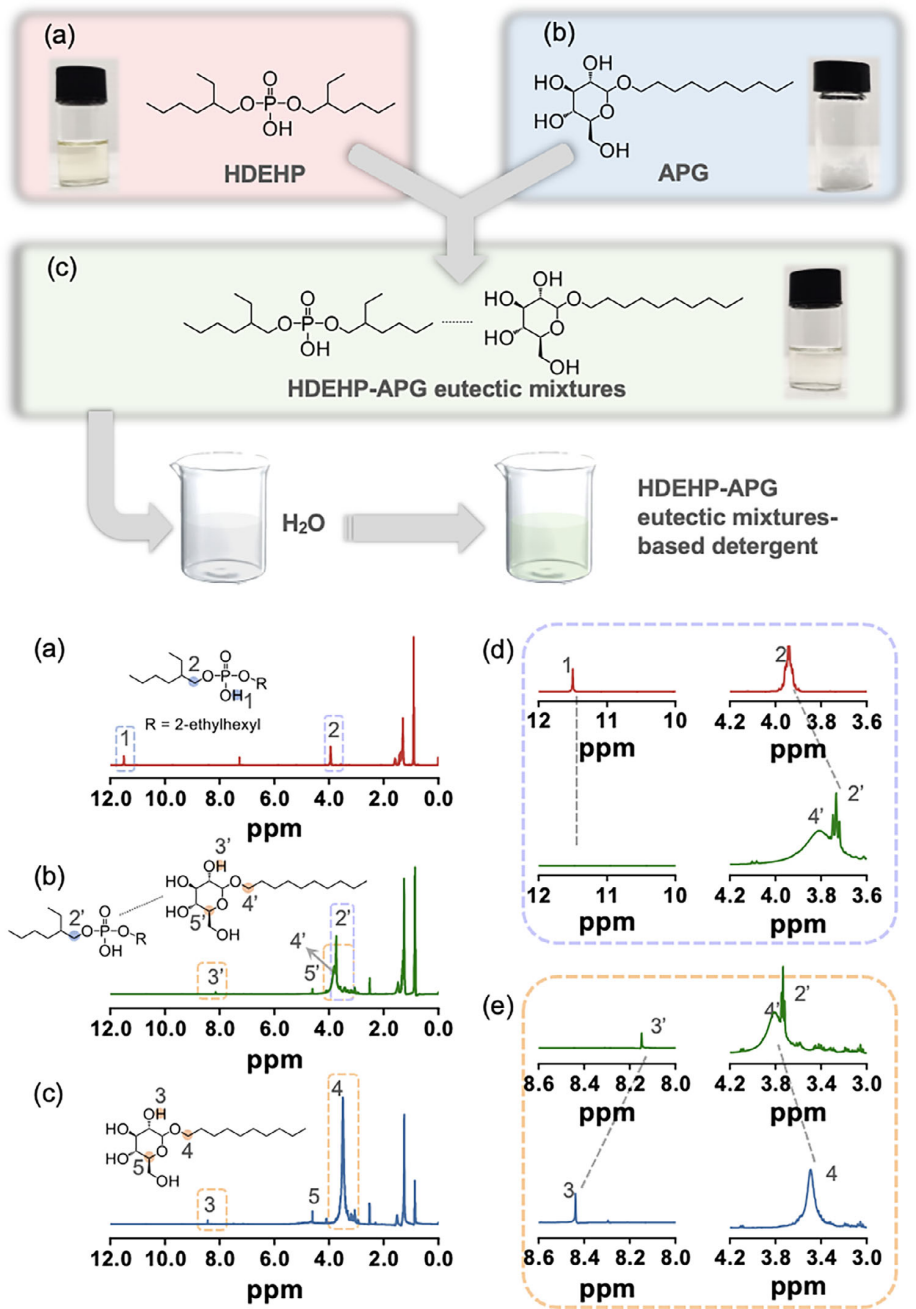
The schematic structures and images of a) HDEHP, b) APG, and c) HDEHP‐APG eutectic mixtures. ^1^H NMR spectra of d) HDEHP, e) HDEHP‐APG eutectic mixtures, and f) APG. g,h) Enlarged ^1^H NMR spectra for comparison.

Consequently, we strategically designed and synthesized innovative eutectic mixtures by integrating a commercially available phosphate compound, bis(2‐ethylhexyl) phosphate (HDEHP), into APG via intermolecular hydrogen bonding interactions (**Figure** **1**a–c). The selection of HDEHP as a modifier is justified by three key factors. 1) The phosphate ligand features a hydrophilic phosphonic acid terminus and two hydrophobic alkyl chains, which can also function as a surfactant to support foam generation. 2) The HO─P and P═O moieties serve as HBD and HBA, respectively, contributing to the stabilization of the detergent's chemical structure through hydrogen bonding. 3) HDEHP has been extensively studied and proven as an effective extractant for Am recovery,^[^ ^43^, ^44^ ^]^ which is desirable for promoting radioactive remediation.

## 2 Results and Discussion

The synthetic strategy was meticulously devised to integrate HDEHP fluid with white powdery APG, resulting in the formation of a colorless HDEHP‐APG liquid product (Figure 1a–c; Supporting Information for details). The observed phase transition of APG suggests that a widely distributed cross‐linked hydrogen bonding network facilitates the aggregation of HDEHP and APG into binary eutectic mixtures. Such intermolecular interactions were subsequently corroborated through an array of characterization techniques, including Nuclear Magnetic Resonance (^1^H NMR, ^13^C NMR) and Fourier Transform Infrared Spectroscopy (FT–IR). Specifically, peaks of *H*O─P and *H*O─CH (functioning as HBD) in ^1^H NMR spectra either disappear or undergo significant shifts upon the formation of HDEHP‐APG eutectic mixtures (signals 1, 3, and 3′, Figure 1d–h).^[^ ^45^, ^46^, ^47^, ^48^ ^]^ Likewise, deviations or deformations are observed in the methylene or methine moieties adjacent to the O atom (acting as HBA) when HDEHP interacts with APG (signals 2, 4, 2′, and 4′, Figure 1d–h).^[^ ^45^, ^46^, ^47^, ^48^ ^]^ These diverse alterations in the H‐signals can be attributed to the extensive hydrogen bonding interactions prevalent within the eutectic mixtures, which are further evidenced by resonance shifts in ^13^C NMR and vibrational changes in FT‐IR spectra, respectively (Figures S1 and S2, Supporting Information).^[^ ^45^, ^46^, ^47^, ^48^ ^]^ Notwithstanding, one attracts our attention is that the structure integrity of the HDEHP‐APG eutectic mixtures may be compromised on the occasion of mixing with water to generate a detergent owing to the pervasive hydrogen bonding interactions within the aqueous environment.^[^ ^49^ ^]^ Therefore, an additional ^1^H NMR analysis of the HDEHP‐APG eutectic mixtures was conducted in a mixed solvent of DMSO‐d + D_2_O, and the results were compared with those obtained in pure DMSO‐d solvent. Remarkably, even with the introduction of excess D_2_O into DMSO‐d, prominent deviations persisted in the peaks corresponding to *H*O─CH, ─C*H*_2_─O─P, and CH─O─C*H*_2_ with respect to APG or HDEHP (signals 2, 3, 4, 2′’, 3′’, and 4′’, Figure S3, Supporting Information). Namely, our afforded eutectic mixtures are sufficiently robust to resist the deconstruction effect by concomitant water.

For a typical APG‐based detergent, APG molecules aggregate into micelle particles and stably disperse in aqueous media.^[^ ^10^ ^]^ Given the amphiphilic nature of HDEHP, characterized by its dual hydrophobic and hydrophilic properties, we hypothesize that the integration of HDEHP could potentially modulate the surface characteristics of APG‐based detergents. To validate this hypothesis, we systematically investigated the interfacial behavior of multicomponent liquid systems (Figure S4, Supporting Information). Obviously, the addition of APG as a surfactant into water substantially diminishes the contact angle between a liquid film and a solid substrate from 57.2° to 31.3° (Figure S4a,b, Supporting Information). Subsequent incremental incorporation of HDEHP further amplifies such a tendency with the contact angle progressively decreasing from 29.1° to 19.3° (Figure S4c–f, Supporting Information). The observed flattening of the liquid film, indicative of reduced interfacial tension, strongly suggests that the formation of eutectic mixtures directly helps to stabilize the structural framework of the detergent.^[^ ^50^ ^]^ Moreover, solutions with a higher proportion of HDEHP show enhanced surface wettability, which significantly improves detergent‐substrate interactions, thereby optimizing the contaminant removal efficiency.

Now that the incorporation of HDEHP remarkably mitigates the stretching force compared to the individual APG system, prompting us to investigate the particle sizes of micelles in both APG‐based and eutectic mixture‐based detergents using dynamic light scattering (DLS). As illustrated in Figure S5 (Supporting Information), when the HDEHP/APG ratio is maintained at or below 1/5, a gradual reduction in particle diameters below 20 nm is observed, concomitant with the emergence of micelles exceeding 100 nm in diameter as the HDEHP proportion increases. Additionally, upon reaching an HDEHP/APG molar ratio of 1/2, the smaller micelles undergo complete disintegration and reassemble into larger aggregates ranging from 60 to 600 nm. Such conversions of the micelles can be attributed to the breakdown of micro‐scale particles and their subsequent reorganization into macro‐scale structures, facilitated by HDEHP acting as a surface modifier. More importantly, the expansion in the particle size not only enhances the stability of detergent, but also suggests promising potential for pollutant encapsulation.

**Figure 2:**
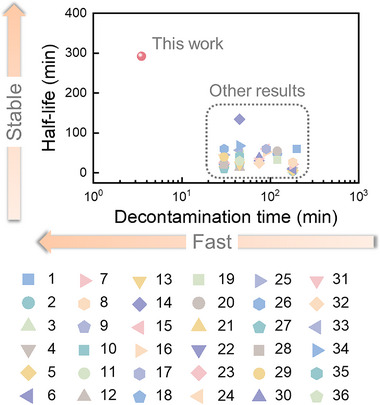
Comparisons with the representative 1/1 HDEHP/APG eutectic mixtures‐based detergent and other detergents in terms of decontamination time and half‐life (Detailed information of the comparative foam detergents see Table S1 (Supporting Information). The numbered labels (1–36) distinguish decontamination systems and are unrelated to citations.).

The stability of foam detergents is conventionally evaluated by their half‐life, defined as the time required for the foam volume to decrease by half.^[^ ^7^, ^8^, ^9^, ^10^, ^14^, ^16^ ^]^ On this account, half‐lives of APG‐based and eutectic mixtures‐based detergents with varying HDEHP/APG mole ratios were determined following foam formation and compared directly (Figure S6a, Supporting Information). Obviously, incorporating HDEHP into APG leads to an upsurge in the stability of foam, as evidenced by the substantial increase in half‐life from 30 min to 150–300 min (Figure S6b, Supporting Information). This enhanced stability facilitates prolonged contact between the detergent and contaminated surfaces, thereby promoting more effective pollutant dissolution. Meanwhile, we observed a corresponding delay in foam drainage with increasing HDEHP content, according with the prior established relationship with interfacial tension. It should be emphasized that the half‐life of the presented eutectic mixtures‐based detergent surpasses that of previously reported decontamination foams (**Figure** **2**). This exceptional stability is closely associated with the synergistic effects of reduced interfacial tension and the formation of extensive internal cross‐linked hydrogen bonding networks among micelles.

**Figure 3:**
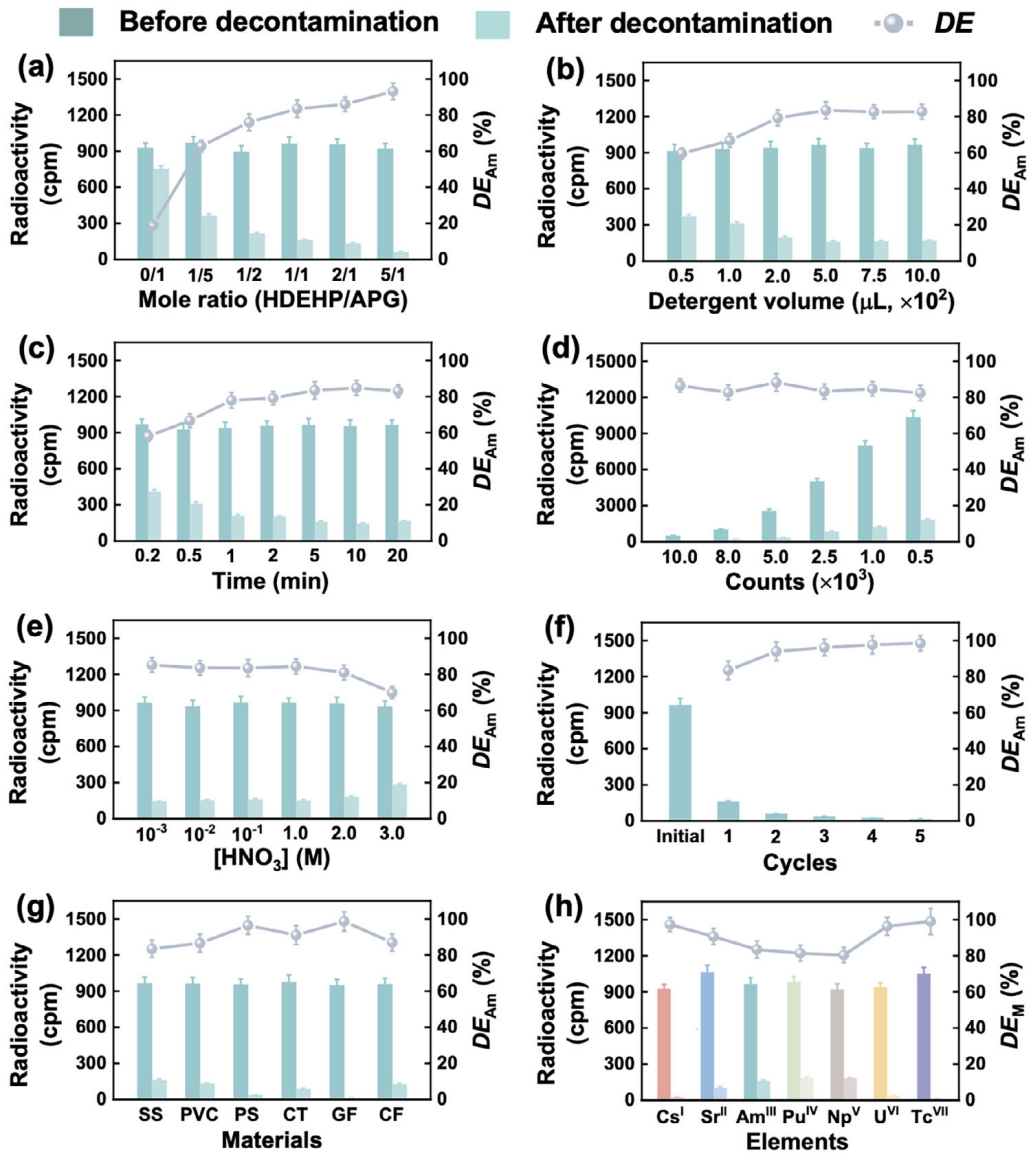
Demonstration of radioactive decontamination. Effects of a) HDEHP/APG mole ratio, b) detergent volume, c) contact time, d) initial contamination content, e) acidity of contaminated liquid, f) decontamination cycle, g) contact material, and h) decontamination element. Experimental conditions. Initial detergent component: (a) APG and HDEHP/APG (1/5‐5/1) or (b–h) HDEHP/APG (1/1) in H_2_O. Initial contaminated liquid: (a–c, e–g) ≈950 cpm Am(III), d) ≈500–10,000 cpm Am(III), and (h) ≈950 cpm of each ion in (a–d,f–h) 0.1 m or (e) 10^−3^‐3.0 m HNO3 solution. Contact material: (a–f,h) stainless‐steel or (g) different materials. Contact time: (a,b, d–h) 5 min or (c) 0.2–20 min. Temperature: 25.0 ± 0.5 °C.

Stainless‐steel (SS) has been widely applied in nuclear facilities manufacturing. Herein, in combination with the optimization of eutectic mixtures‐based detergent in terms of wettability and stability, one can treat a stainless‐steel surface contaminated with Am utilizing this specially designed chemical agent. Evidently, the incorporation of HDEHP into APG brings about a forward leap in single‐stage Am decontamination efficiency (*DE*_Am_), with an increase of over 300% (from 19% of APG‐based detergent to 83% of 1/1 HDEHP/APG‐based detergent, **Figure** **3**a). This advancement addresses the previously unmet challenge of removing strong α‐radioactive nuclides using foam detergents. Meanwhile, *DE*_Am_ can be further improved by appropriately increasing detergent consumption (Figure 3b). Excitedly, the improved wettability does prominently accelerate the decontamination process as expected, with the foam's residence time on the contaminated surface being a mere 2–5 min (Figure 3c), substantially shorter than that of other systems (Figure 2). It is also noteworthy that the decontamination efficiency of Am remains largely unaffected by the initial dose of radioactive contamination, thereby enhancing the practical applicability of our detergent in real scenarios (Figure 3d). *DE*_Am_ value decreases slightly to ≈70% (Figure 3e) in the case of acidity of the contaminated solution increasing to 3.0 m, which can be ascribed to acid‐induced corrosion of the iron surface and concomitant Am permeation. Notwithstanding, this stubborn contamination can be quantitatively removed through multiple operational cycles (Figure 3f). A further investigation of various contaminated substrates demonstrates that the decontamination efficiencies exceed 90% for painted surfaces (PS), ceramic tiles (CT), and glass flakes (GF). Moreover, pollutant removal from polyvinyl chloride plastic (PVC) and cement flakes (CF) surfaces is significantly more efficient than that from stainless‐steel (SS) (Figure 3g). Such discrepancies likely arise from the acid‐resistant nature of non‐SS materials, resulting in weakly physical absorption of Am by PVC and CF, and even less interaction on the smoother PS, CT, and GF surfaces. Given the effective decontamination of Am, we subsequently explored the removal of representative radionuclides with thermodynamically stable oxidation states ranging from +I to +VII (cesium, Cs(I); strontium, Sr(II); Am(III); plutonium, Pu(IV); neptunium, Np(V); uranium, U(VI); technetium, Tc(VII)).^[^ ^51^, ^52^, ^53^ ^]^ Surprisingly, exceptional decontamination efficiencies over 80% were achieved for all the concerned ions, with *DE* values for Cs(I), Sr(II), U(VI), and Tc(VII) surpassing 90% (Figure 3h). This unprecedented and extensive decontamination performance has sparked our keen interest in further elucidating the underlying mechanisms.

The interaction between the afforded detergent and metal ions was preliminary inspected through absorption spectroscopy. In consideration of the extremely intense radioactivity associated with macro amounts (≈mm level) of Am, neodymium (Nd) was employed as an analog owing to the equivalent trivalent state, comparable ionic radius,^[^ ^37^, ^38^ ^]^ and absorption characteristics in the visible light region.^[^ ^54^, ^55^, ^56^ ^]^ As depicted in Figure S8a (Supporting Information), persistent addition of HDEHP into a mixture containing a fixed quantity of Nd(III) and APG resulted in a demonstrable decrease in the absorption spectra. Conversely, maintaining constant concentrations of Nd(III) and HDEHP while increasing the molar equivalent of APG did not induce significant alterations in the absorption band (Figure S8b, Supporting Information). The striking contrast spectral changes underscore robust complexation capability of HDEHP toward trivalent metal ions, a property not shared by APG. Such an interaction was further validated by NMR spectroscopy. To circumvent interference from the paramagnetic nature of Nd(III), different molar proportions of diamagnetic lanthanum (La(III)), serving as a surrogate for Am(III),^[^ ^56^, ^57^ ^]^ were introduced into the eutectic mixtures. The signal of C*H*_2_‐O‐P undergoes a regular shift in the course of interacting with La(III) (Figure S9, Supporting Information), which is in line with prior findings in absorption spectra. As a consequence, the intense chemical complexation by HDEHP in our presented system, as opposed to the feeble physical adsorption typical of conventional foam detergents, significantly advances the efficacy of Am decontamination.

**Figure 4:**
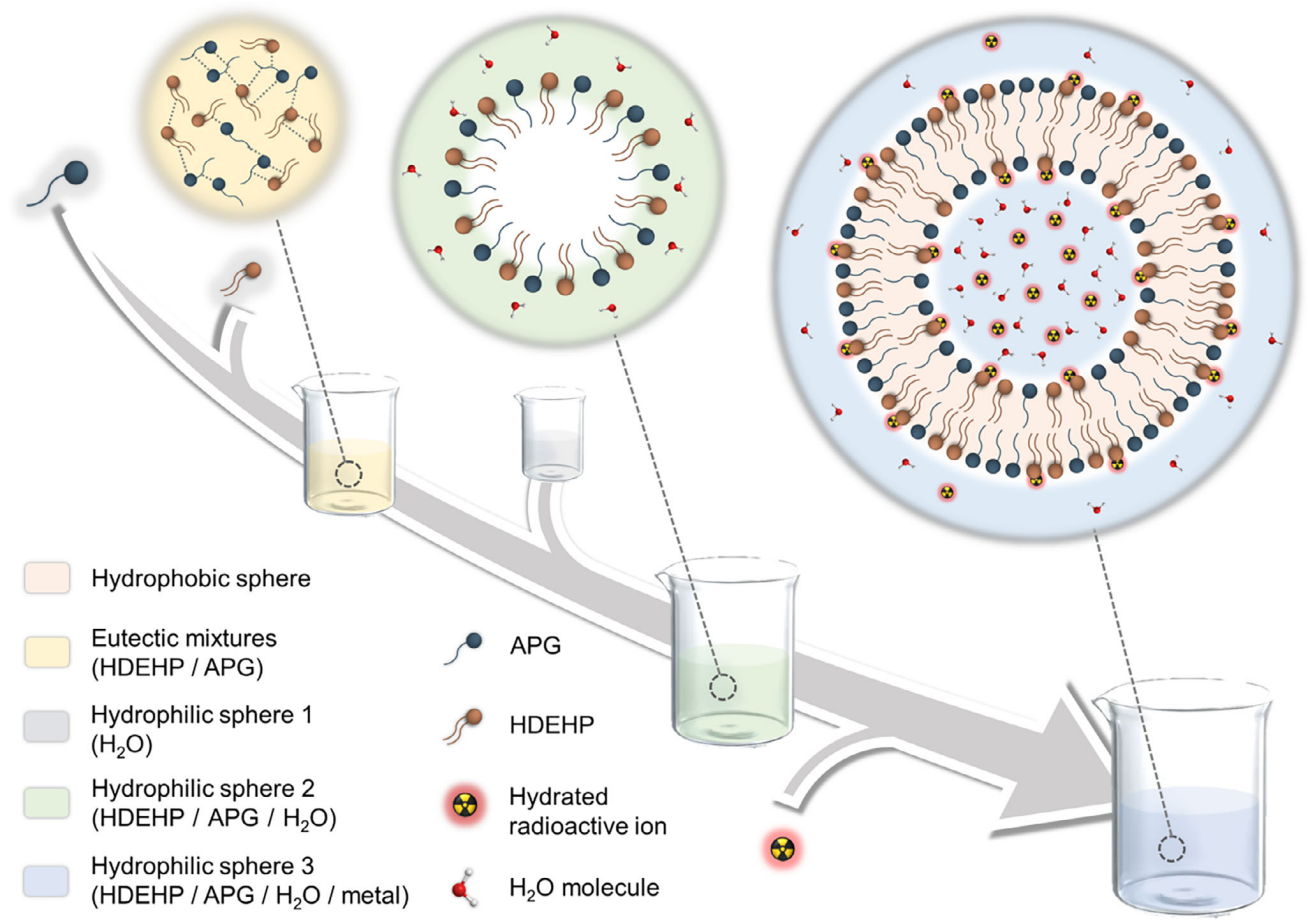
Diagram of decontamination mechanism.

The dimensions of micelle particles in aqueous solution were further determined following the incorporation of eutectic mixtures‐based detergent with varying proportions of Nd(III). A pronounced enlargement of the particles was observed with increasing Nd(III) concentration (Figure S10a, Supporting Information), indicative of the substantial encapsulation of hydrated metal ions within the micelles. This process facilitates the transformation of initial monomolecular layer micelles into vesicular state bimolecular layer structures, driven by the entrapment of water molecules and hydrated ions (**Figure** **4**). This mechanism markedly contrasts with the conventional decontamination paradigm, which relies on foam adsorption at the gas‐liquid interface.^[^ ^5^, ^6^, ^7^, ^8^, ^9^, ^10^, ^13^, ^17^, ^21^ ^]^ To corroborate this view, the particle sizes were assessed upon mixing the detergent with ions exhibiting diverse oxidation states. Notably, HDEHP‐APG mixtures accompanied with high‐charge‐density ions including thorium, (Th(IV)), U(VI) and Nd(III) grew as more macroscopic particles than those containing low‐charge‐density cations like Cs(I) and Sr(II) (Figure S10b, Supporting Information), which can be attributed to the enhanced attraction of H_2_O molecules by the high‐charge‐density ions in the aqueous solution.^[^ ^58^, ^59^, ^60^, ^61^, ^62^, ^63^ ^]^ Accordingly, when contaminants are encapsulated by the eutectic mixtures‐based detergent, the charged ions bring considerable amounts of water into the vesicular architecture, thereby facilitating the formation of relatively larger volumetric particles.

The process of preceding decontamination can be schematically depicted as Figure 4. Initially, HDEHP, serving as a surface modifier, is integrated into APG to form eutectic mixtures, which are stabilized by an extensive network of cross‐linked hydrogen bonds (indicated by the yellow circle). Subsequently, these HDEHP‐APG eutectic mixtures are introduced into water, where they spontaneously assemble into monomolecular layer micelles (highlighted by the green circle). Such micelles possess superior wettability and stability compared to the individual APG‐based structures. Upon contact with a contaminated surface, the eutectic mixtures‐based detergent facilitates two key interactions: first, HDEHP within the structure forms strong chemical bonds with metal ions, and second, the monomolecular layer micelles undergo a conversion into bimolecular layer vesicular structures, encapsulating hydrated ions within (denoted by the blue circle). Consequently, the synergistic effects of complexation and encapsulation intrinsically boost efficient decontamination of contaminated metal ions.

## 3 Conclusion

In summary, we have successfully integrated HDEHP into APG to synthesize novel eutectic mixtures, which spontaneously assemble into micelles in aqueous solution. The extensive hydrogen bonding network significantly enhances the surface wettability and stabilizes the structural integrity of the foam detergent. Furthermore, an unprecedented decontamination efficiency for Am and multivalent ions has been achieved through a unique synergistic complexation‐encapsulation strategy, thereby paving the way for the development of more efficient radioactive detergents in the future. Beyond its immediate applications, this work highlights the untapped potential of eutectic solvents in interfacial engineering and functional material design, opening new avenues for advanced material development.

## Conflict of Interest

The authors declare no conflict of interest.

## Supporting information



Supporting Information

Supporting Information

## Data Availability

The data that support the findings of this study are available from the corresponding author upon reasonable request.
