# Supplementary material for: A Synergistic Complexation‐Encapsulation Paradigm Enables Unprecedented Radioactive Remediation
Source: Adv Sci (Weinh). 2025 Oct 3;12(48):e04940. doi: 10.1002/advs.202504940 (PMC12752549; doi:10.1002/advs.202504940)
Supplement: Supplementary file 1 — Supporting Information [file ADVS-12-e04940-s001.docx]

**Supporting Information**

**A Synergistic Complexation-Encapsulation Paradigm Enables Unprecedented Radioactive Remediation**

Qilong Tang^1^, Huaixin Hao^1^, Xue Dong^2^, Chao Xu^1,*^, and Zhipeng Wang^1,*^

^1^ Institute of Nuclear and New Energy Technology, Tsinghua University, Beijing 100084, China
^2^ The 404 Company Limited, China National Nuclear Corporation, Lanzhou 732850, China

**Contents**

[**1. Experimental and Measurements S1**](#_Toc204797739)

[**1.1. Chemicals and materials S1**](#_Toc204797740)

[**1.2. Syntheses of eutectic mixtures and foam detergent S1**](#_Toc204797741)

[**1.3. Characterizations S1**](#_Toc204797742)

[**1.4. DLS measurements S2**](#_Toc204797743)

[**1.5. Absorption spectra analyses S2**](#_Toc204797744)

[**1.6. Radioactive decontamination S2**](#_Toc204797745)

[**1.7. Statistical Analysis S3**](#_Toc204797746)

[**2. Supplementary Figures (Figure S1 to Figure S10) S4**](#_Toc204797747)

[**3. Supplementary Table (Table S1) S10**](#_Toc204797748)

[**4. References S11**](#_Toc204797749)

2. Experimental and Measurements
   1. Chemicals and materials

^137^Cs, ^90^Sr, ^241^Am, ^239^Pu, ^237^Np, ^233^U and ^99^Tc provided by the Institute of Nuclear and New Energy Technology (INET), Tsinghua University, were used as radioactive tracers in decontamination experiments. Macro amount of Nd(NO_3_)_3_, La(NO_3_)_3_, ^235,238^UO_2_(NO_3_)_2_ and ^232^Th(NO_3_)_4_ were applied in absorption spectra, NMR and DLS tests. The analytical-grade HDEHP and APG (Figure 1) were purchased from Macklin Chemical Technology Co. Ltd. Deuterated reagents of DMSO-d and D_2_O were obtained from J&K Scientific Co. Ltd. Milli-Q water was used for all the experiments. Tetramethylsilane (TMS) was the internal standard substance for ^1^H NMR. Unless specifically noted, all the other reagents involved in this work were of AR grade or higher quality and used without further purification. CAUTION: *^137^Cs, ^90^Sr, ^99^Tc are intense β/γ emitters and ^241^Am, ^239^Pu, ^237^Np, ^233^U possess high α radiotoxicity, which impose serious health threat and the relevant experiments must be conducted in a specifically designed fume hood for radionuclides operations.*

- 1. Syntheses of eutectic mixtures and foam detergent

HDEHP-APG eutectic mixtures (mole ratio = 1/1). Firstly, 3.22 g of HDEHP liquid (10 mM) was dropped in 20 mL anhydrous methanol to provide a transparent homogeneous solution. Afterwards, 3.20 g of white powdery APG (10 mM) was also dissolved in equal volume anhydrous methanol, obtaining a colorless solution. Subsequently, HDEHP-containing solution was added dropwise into APG system with magnetic stirring. Finally, over a period of 1 h mingling, the solution was concentrated by reduced pressure distillation to afford colorless fluid remnant (6.40 g, yield: 99.69%).

Foam detergent. Mixing the synthesized colorless fluid into deionized water with a mass ratio of 1:99 to generate a slightly emulsified solution as eutectic mixtures-based foam detergent.

The detergents with different HDEHP/APG mole ratios can also be prepared according to the above-described procedures.

- 1. Characterizations

NMR. NMR spectra were recorded on Bruker Avance III Model 600 MHz instrument (Bruker, Inc.). 5-10 mg samples of HDEHP, APG and eutectic mixtures were dissolved in DMSO-d or DMSO-d + D_2_O (v/v = 1/1) mixed solvent for ^1^H NMR and ^13^C NMR tests. The La(III)/HDEHP/APG mixtures with different mole ratios were dissolved in DMSO-d for ^1^H NMR spectra collection.

FT-IR. 20 mg samples of HDEHP, APG and eutectic mixtures were determined on Nicolet iZ10 Spectrophotometer (Thermo Fisher Scientific, Inc.).

Contact angle. The contact angles of H_2_O, APG-H_2_O solution and eutectic mixtures-containing aqueous solutions were detected with an optical contact angle measuring and contour analysis system (OCAH 200, Dataphysics Instruments).

- 1. DLS measurements

DLS measurements of the aqueous solutions were performed on a Malvern Nano ZS90 instrument (Malvern Instruments) at 298.0 ± 0.1 K with a scattering angle of 90^°^. In a typical operation, 2 mL of APG-containing or HDEHP/APG-containing or metal ion/HDEHP/APG-containing aqueous solutions were added into a 10 mm cuvette with four optical paths and then fixed on the size matched cuvette chamber. Data were then collected after stabilization of the solution (10 min).

- 1. Absorption spectra analyses

Absorption spectra of aqueous solutions containing Nd(III) or Nd(III)/HDEHP/APG mixtures with different mole ratios were collected on Cary 6000i UV-Vis-NIR spectrophotometer (Agilent Inc.). Specifically, the aqueous solution was added into a 10 mm cuvette with screwed cap. H_2_O was designated as the reference. The absorption spectra were collected in the wavelength range of 560-600 nm owing to the characteristic peak of Nd(III) within this region. The temperature of the sample and reference cell holders was controlled as 298.0 ± 0.1 K via an external temperature controller, which drives a Peltier heat pump attached to holders.

- 1. Radioactive decontamination

The operations of radioactive decontamination are shown in Figure S7 and mainly summarized as seven steps. 1. Using a NuviaTech Healthcare CoMo 170 contamination monitor (NuviaTECH Instruments) with α-ray and β/γ-ray identification function to detect the radioactivity of uncontaminated stainless steel plate. The apparatus was placed on a specially customized stainless steel shelf to ensure stability and comparability for each measurement. The radioactive background was recorded as A_0_. 2. Appropriate radionuclide-containing solution was sampled by a pipette and injected onto the stainless steel plate and then placed overnight for air drying. 3. The contaminated plate was measured and the radioactivity was recorded as A_1_. 4. Foaming by sufficient shaking of the prepared detergent. 5. The foam was added on the contaminated plate and stood for a period for radioactive decontamination. 6. Cleaning the surface by gently wiping. 7. The decontaminated plate was thereafter detected and the radioactivity was recorded as A_2_. The afore-mentioned operations are also applicable to the decontamination of surfaces made of other materials.

The net radioactivity of contaminated and decontaminated plates was calculated as (A_1_ - A_0_) and (A_2_ - A_0_), respectively. The decontamination efficiency (*DE*) of a specific radionuclide (M) was defined as the quotient of removed radioactivity (A_1_ - A_2_) to that of net contaminated radioactivity (A_1_ - A_0_), *DE*_M_ = (A_1_ - A_2_)/(A_1_ - A_0_).

- 1. Statistical Analysis

All decontamination experiments were independently repeated at least three times (n = 3), and the results are presented as mean ± standard deviation (SD). Error bars in the figures represent SD. No data transformation or outlier exclusion was performed. No statistical significance testing was applied in this study. All statistical analyses and graphical presentations were conducted using OriginPro 2024.

1. Supplementary Figures (Figure S1 to Figure S10)


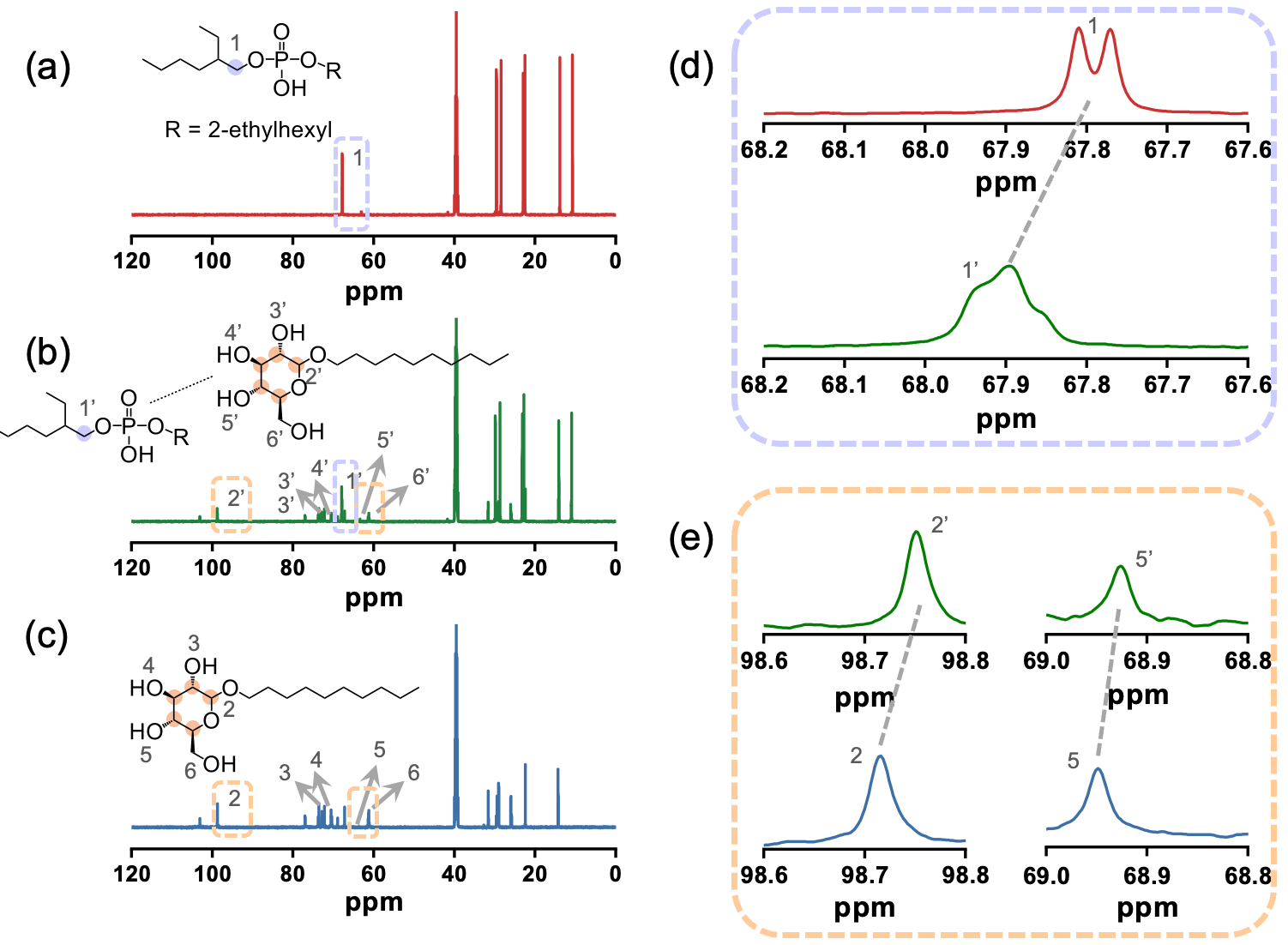


**Figure S1.** ^13^C NMR spectra of (a) HDEHP, (b) HDEHP-APG eutectic mixtures and (c) APG. (d,e) Enlarged ^13^C NMR spectra for comparison.


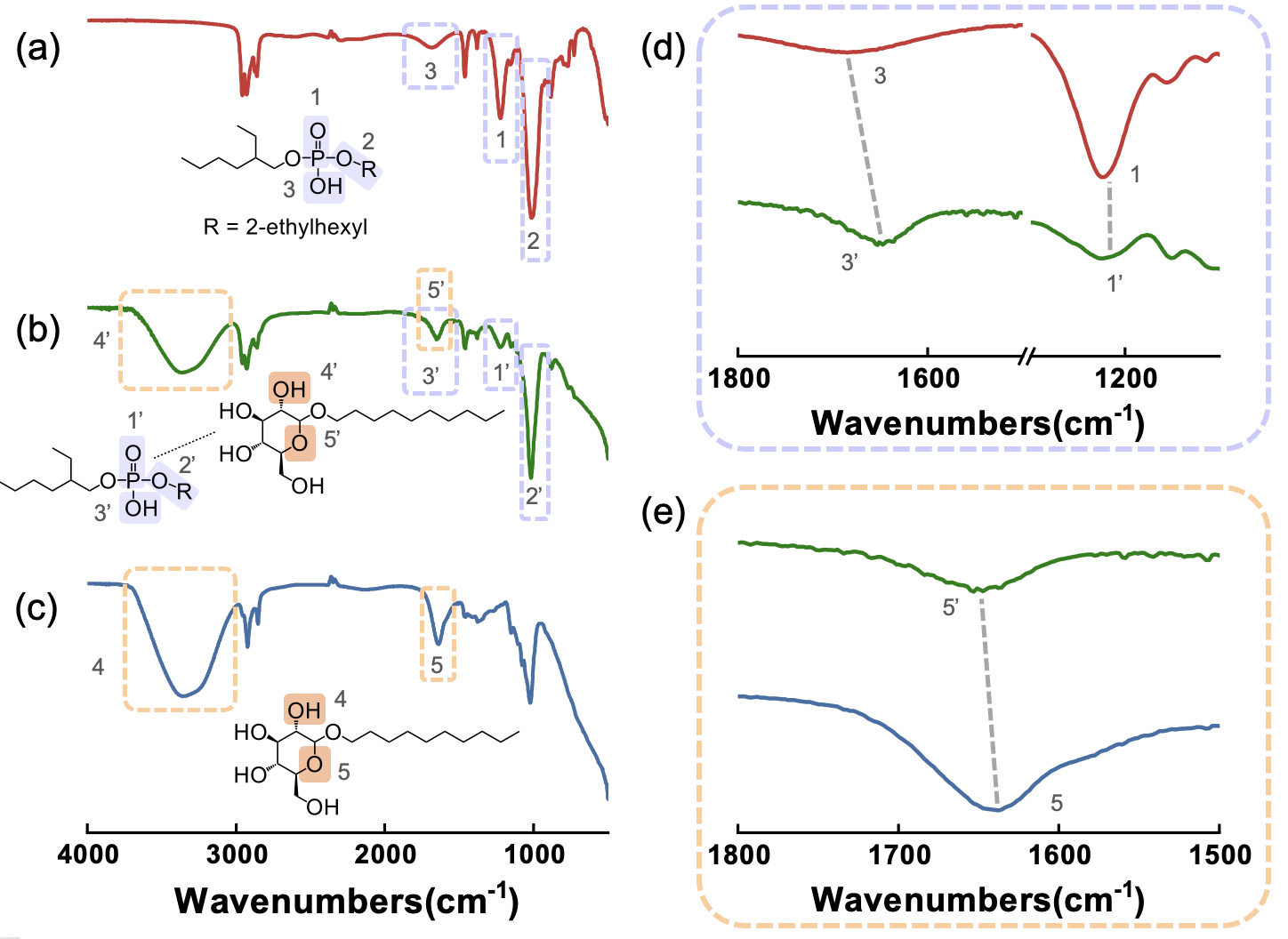


**Figure S2.** FT-IR spectra of (a) HDEHP, (b) HDEHP-APG eutectic mixtures and (c) APG. (d,e) Enlarged FT-IR spectra for comparison.


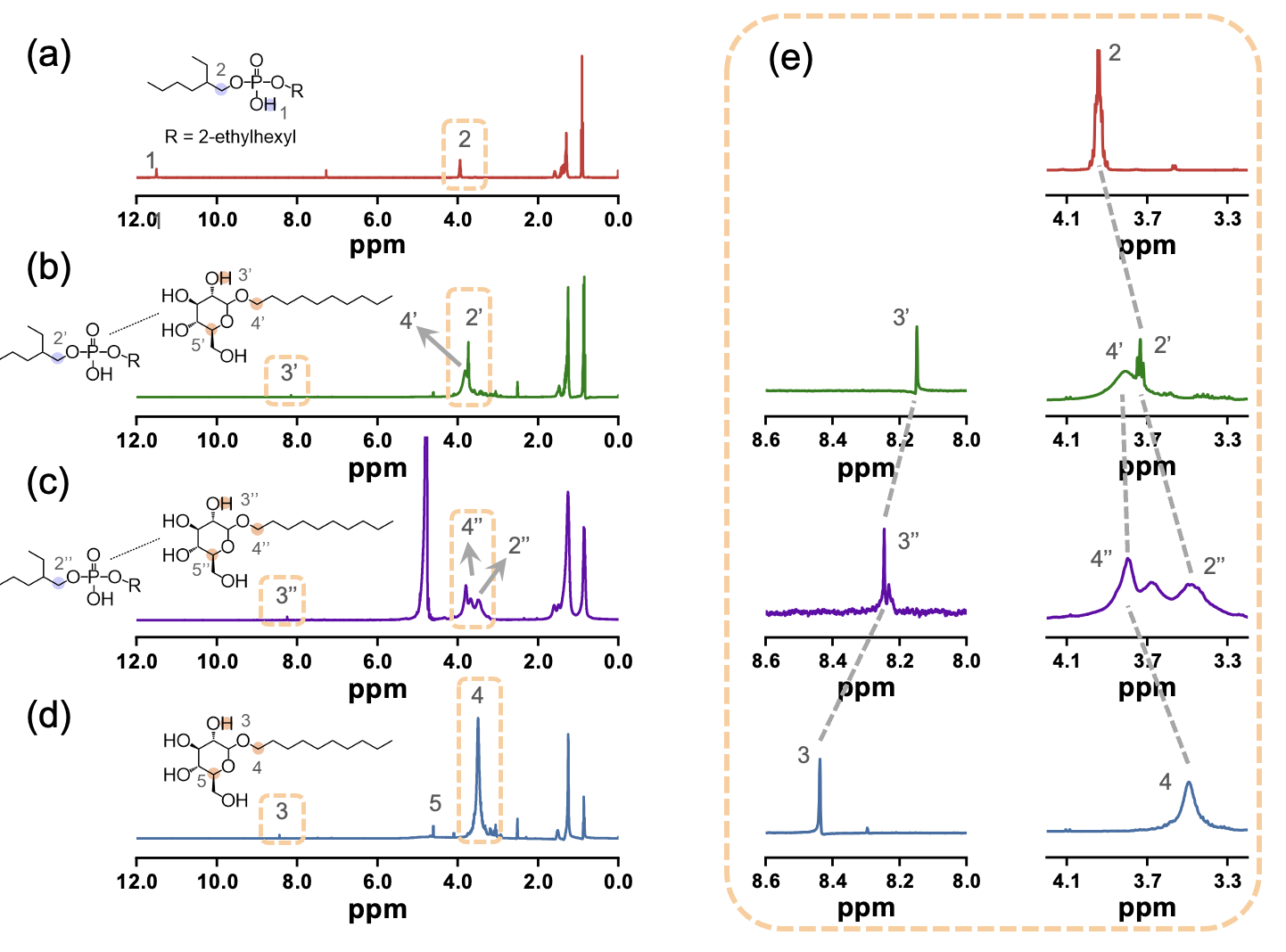


**Figure S3.** ^1^H NMR spectra of (a) HDEHP, (b,c) HDEHP-APG eutectic mixtures and (d) APG. (e) Enlarged ^1^H NMR spectra for comparison. Solvents for ^1^H NMR determination in spectra (a,b,d) and (c) are DMSO-d and DMSO-d + D_2_O mixture, respectively.


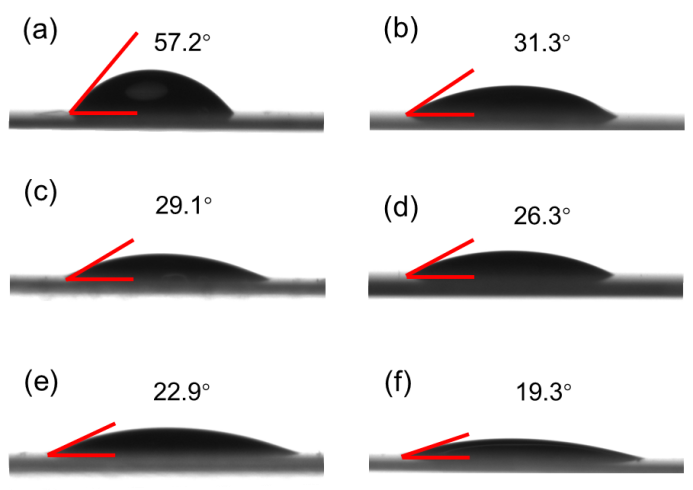


**Figure S4.** Contact angles of (a) H_2_O, (b) APG-H_2_O solution and (c-f) eutectic mixtures-containing aqueous solutions with HDEHP/APG mole ratios of (c) 1/20, (d) 1/10, (e) 1/5 and (f) 1/2.


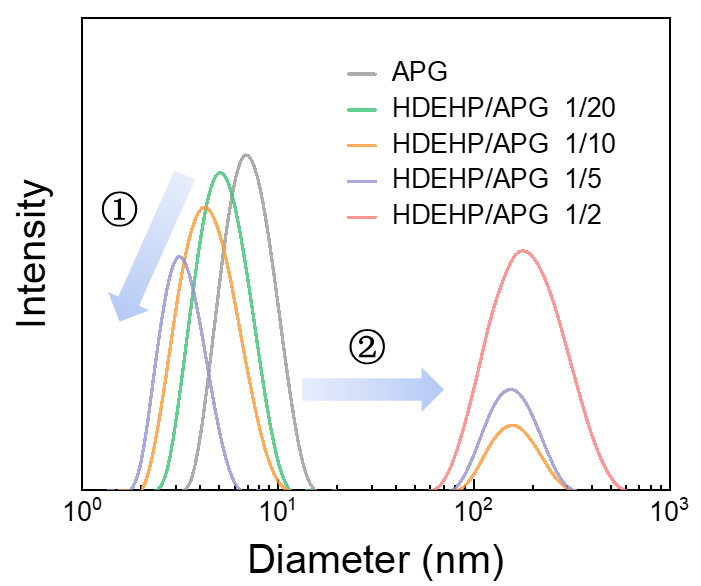


**Figure S5.** Sizes of micelle particles in APG-based detergent and eutectic mixtures-based detergents with HDEHP/APG mole ratios of 1/20, 1/10, 1/5 and 1/2.


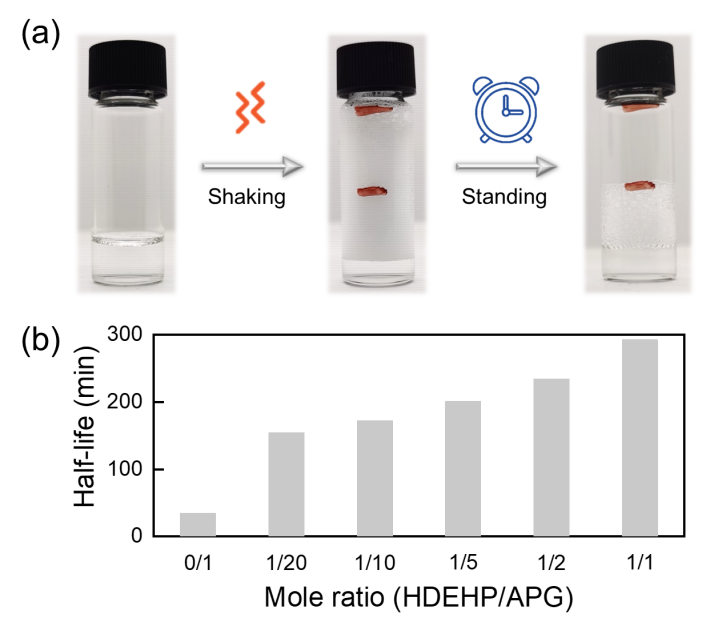


**Figure S6.** (a) Diagram of the processes of foaming by shaking detergent solution and defoaming by solution standing. (b) Half-lives of different foam detergents.


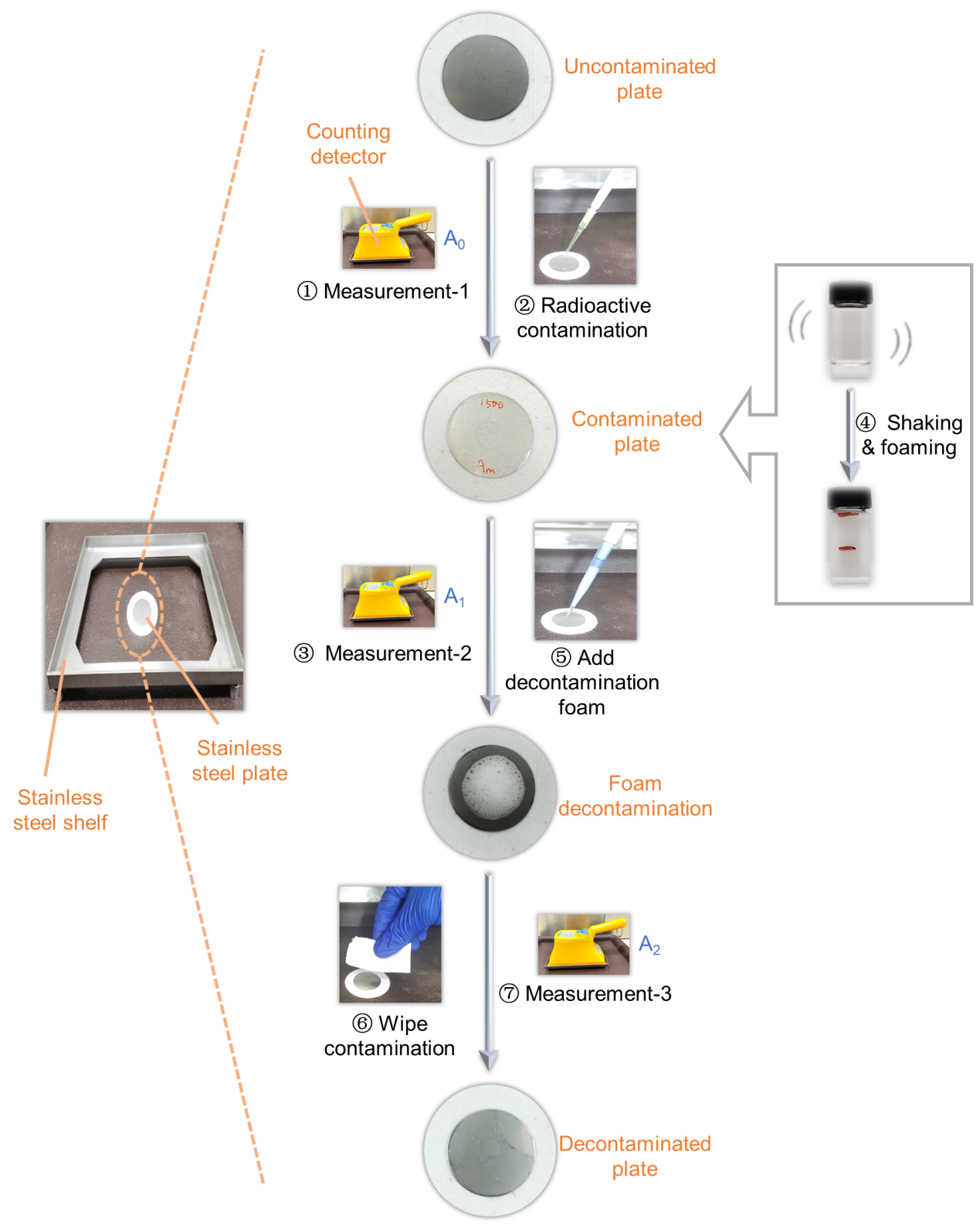


**Figure S7.** Diagram of radioactive contamination, decontamination and measurement operations.


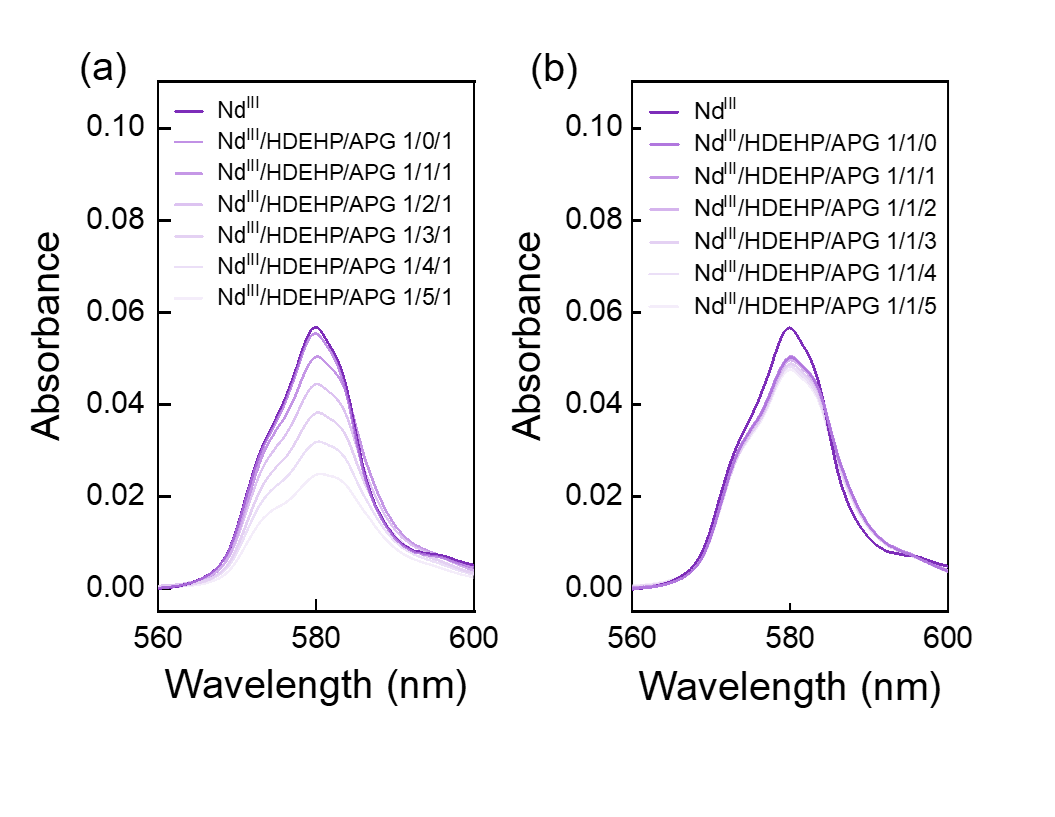


**Figure S8.** Normalized absorption spectra of Nd(III) and Nd(III)/HDEHP/APG mixtures. (a) Quantitative of Nd(III) and APG with variable equivalent of HDEHP; (b) Quantitative of Nd(III) and HDEHP with variable equivalent of APG.


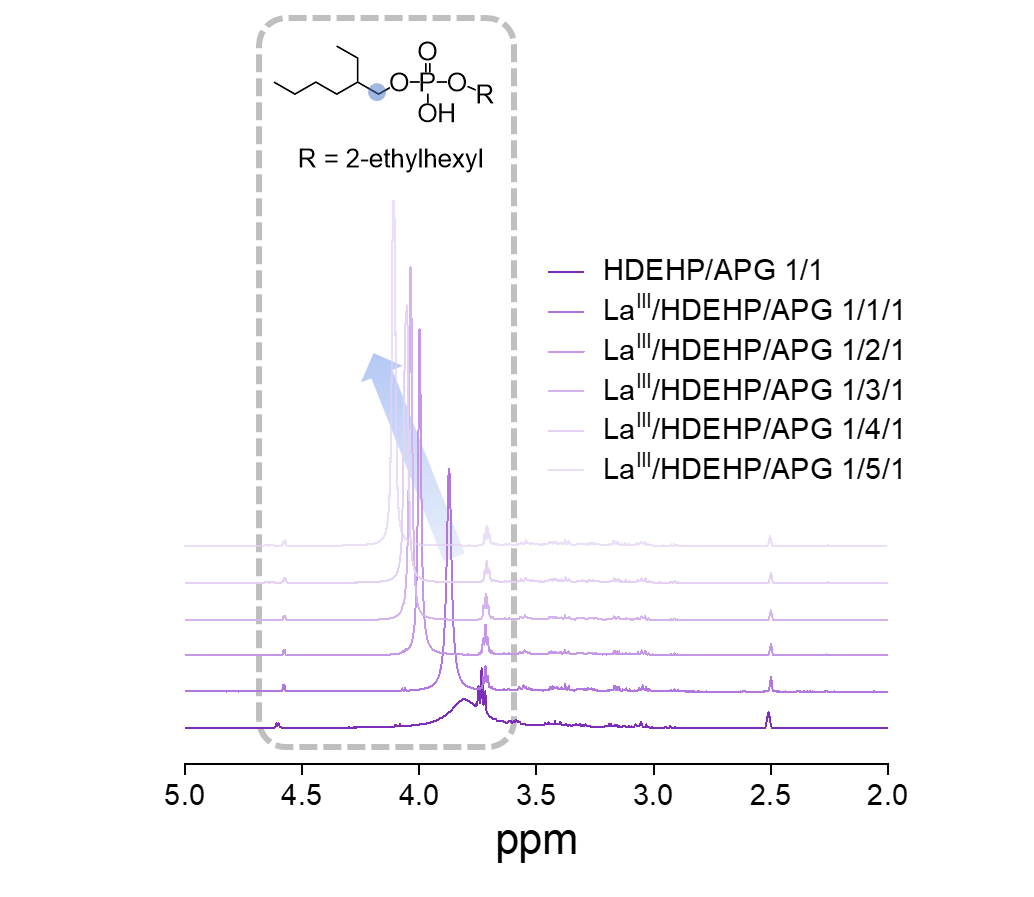


**Figure S9.** ^1^H NMR spectra of HDEHP/APG eutectic mixtures and La(III)/HDEHP/APG mixtures. Quantitative of La(III) and APG with variable equivalent of HDEHP.


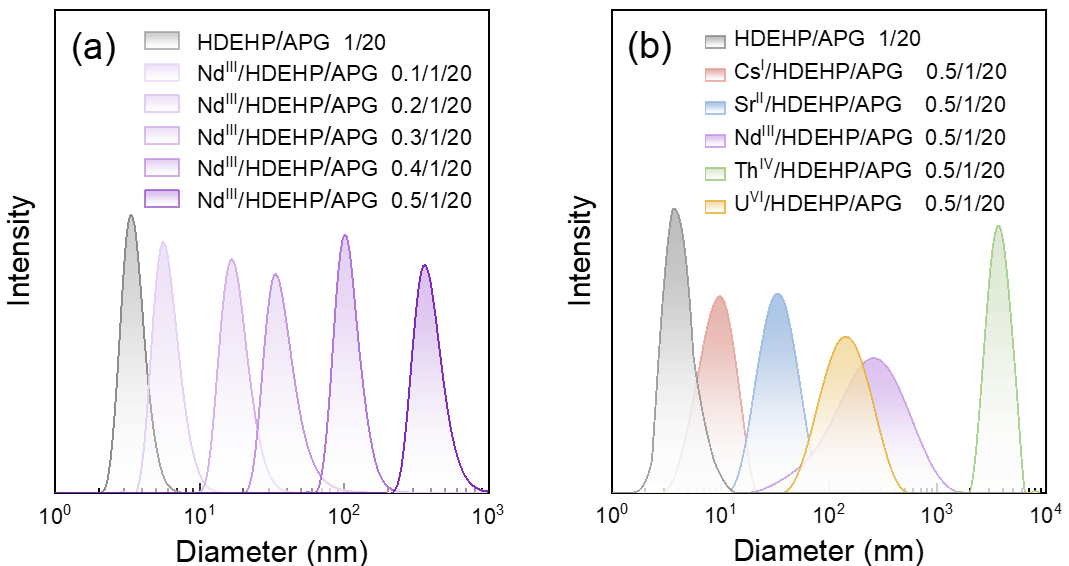


**Figure S10.** Variations in particle size of HDEHP/APG eutectic mixtures (1/20) mixing with (a) different concentrations of Nd(III) or (b) representative ions with multiple oxidation states.

1. Supplementary Table (Table S1)

**Table S1.** Information of the comparative foam detergents.

| **No.** | **Components** | **Ref.** |  | **No.** | **Components** | **Ref.** |
| --- | --- | --- | --- | --- | --- | --- |
| 1 | TBP-PU | [1] |  | 19 | Alk-MXene | [2] |
| 2 | SN-SDS | [3] |  | 20 | Alk-MXene@ODA | [2] |
| 3 | SMS-NPs | [4] |  | 21 | TAS | [2] |
| 4 | CMS-NPs | [4] |  | 22 | APG | [2] |
| 5 | M-5-NPs | [4] |  | 23 | TAS/APG | [2] |
| 6 | SDS-NPs | [5] |  | 24 | TAS/APG/WPU | [2] |
| 7 | CTAB-NPs | [5] |  | 25 | TAS/APG/WPU/TA | [2] |
| 8 | TBS-NPs | [5] |  | 26 | AOS-LA | [6] |
| 9 | APG-AOS-CA-KGM | [7] |  | 27 | DA | [8] |
| 10 | APG-AOS-CA-CMC-Na | [7] |  | 28 | TD | [8] |
| 11 | APG-AOS-CA-XG | [7] |  | 29 | OD | [8] |
| 12 | APG-AOS-CA-EG-KGM | [7] |  | 30 | NS | [9] |
| 13 | APG-AOS-CA-EG-CMC-Na | [7] |  | 31 | XG | [9] |
| 14 | APG-AOS-CA-EG-XG | [7] |  | 32 | TD | [9] |
| 15 | Amine-NPs | [10] |  | 33 | TAS-SiO_2_ | [11] |
| 16 | Thiol-NPs | [10] |  | 34 | TAS-SiO_2_-GO-OH | [11] |
| 17 | Propyl-NPs | [10] |  | 35 | TAS-GO-OH | [11] |
| 18 | MXene | [2] |  | 36 | TAS-GO | [11] |

1. References

[1] S. V. S. Rao, K. B. Lal, S. V. Narasimhan, J. Ahmed. Separation of cobalt from synthetic intermediate and decontamination radioactive wastes using polyurethane foam. *Sep. Sci. Technol.* **1997**, *32* (18), 3007, <https://doi.org/10.1080/01496399708000792>.

[2] C. H. Chen, H. L. Xi, X. Y. Lin, Y. Wang, Z. G. Li. Study on modified MXene to increase the stability and decontamination properties of biomass-based antifreeze foam detergent. *Colloid Surf. A-Physicochem. Eng. Asp.* **2022**, *653*, 130014, <https://doi.org/https://doi.org/10.1016/j.colsurfa.2022.130014>.

[3] I. H. Yoon, C. H. Jung, S. B. Yoon, S. Y. Park, J. K. Moon, W. K. Choi. Effect of silica nanoparticles on the stability of decontamination foam and their application for oxide dissolution of corroded specimens. *Ann. Nucl. Energy* **2014**, *73*, 168, <https://doi.org/https://doi.org/10.1016/j.anucene.2014.06.046>.

[4] I. H. Yoon, S. B. Yoon, C. H. Jung, C. Kim, S. Kim, J. K. Moon, W. K. Choi. A highly efficient decontamination foam stabilized by well-dispersed mesoporous silica nanoparticles. *Colloid Surf. A-Physicochem. Eng. Asp.* **2019**, *560*, 164, <https://doi.org/https://doi.org/10.1016/j.colsurfa.2018.10.002>.

[5] I.-H. Yoon, S. E. Kim, M. Choi, S. Kim, W.-K. Choi, C.-H. Jung. Highly enhanced foams for stability and decontamination efficiency with a fluorosurfactant, silica nanoparticles, and Ce(IV) in radiological application. *Environ. Technol.* **2020**, *18*, 100744, <https://doi.org/https://doi.org/10.1016/j.eti.2020.100744>.

[6] H. Zhang, H. Xi, X. Lin, L. Liang, Z. Li, X. Pan, X. Luo. Biodegradable antifreeze foam stabilized by lauryl alcohol for radioactive surface decontamination. *J. Radioanal. Nucl. Chem.* **2022**, *331* (7), 3135, <https://doi.org/10.1007/s10967-022-08349-3>.

[7] H. Zhang, H. L. Xi, Z. G. Li, X. H. Pan, Y. Wang, C. H. Chen, X. Y. Lin, X. G. Luo. The stability and decontamination of surface radioactive contamination of biomass-based antifreeze foam. *Colloid Surf. A-Physicochem. Eng. Asp.* **2021**, *624*, 126774, <https://doi.org/https://doi.org/10.1016/j.colsurfa.2021.126774>.

[8] H. Zhang, L. Liang, H. Xi, D. Liu, Z. Li, X. Lin. Effects of Fatty Alcohols with Different Chain Lengths on the Performance of Low pH Biomass-Based Foams for Radioactive Decontamination. *Molecules* **2022**, *27* (19), 6627, <https://doi.org/10.3390/molecules27196627>.

[9] H. Zhang, L. Liang, H. Xi, X. Lin, Z. Li, Y. Jiao. Effects of Different Types of Stabilizers on the Properties of Foam Detergent Used for Radioactive Surface Contamination. *Molecules* **2023**, *28* (16), 6107, <https://doi.org/10.3390/molecules28166107>.

[10] I. H. Yoon, S. B. Yoon, Y. Sihn, M. S. Choi, C. H. Jung, W. K. Choi. Stabilizing decontamination foam using surface-modified silica nanoparticles containing chemical reagent: Foam stability, structures, and dispersion properties. *RSC Adv.* **2021**, *11* (3), 1841, <https://doi.org/10.1039/D0RA07644A>.

[11] D. T. Liu, S. J. Lei, Y. Hu, Z. G. Li, H. L. Xi, X. Y. Lin. Environmentally friendly tea saponin foam detergents costabilized by GO-OH/SiO2 nanoparticles for removing radioactive surface contaminants. *Colloid Surf. A-Physicochem. Eng. Asp.* **2024**, *680*, 132667, <https://doi.org/https://doi.org/10.1016/j.colsurfa.2023.132667>.
